# Predicting Self-Rated Health in Diabetes and Chronic Heart Failure – A Multiple Mediation Model

**DOI:** 10.3389/fpubh.2015.00266

**Published:** 2015-11-25

**Authors:** Sylvia Böhme, Babette Renneberg

**Affiliations:** ^1^Department of Psychology, Freie Universität Berlin, Berlin, Germany

**Keywords:** self-rated health, functional health, well-being, comorbidity, multimorbidity, chronic disease

## Abstract

**Purpose:**

Self-rated health (SRH) is a powerful predictor of health-related outcomes such as morbidity and mortality. Aim of the current study was to examine the role of comorbidity, well-being, functional health, and physical limitations as possible predictors of SRH in diabetes and chronic heart failure (CHF).

**Methods:**

Three large samples with persons suffering from diabetes (*n* = 974), CHF (*n* = 955), or both diseases combined (*n* = 934) were analyzed longitudinally over the course of 4 months. To test the mediating effect of comorbidity, well-being, functional health, and physical limitations in association with former and future SRH multiple mediator models were applied.

**Results:**

Across all groups emotional well-being was a consistent and powerful determinant of SRH. The effects of functional health and physical limitations on SRH were also significant but varied between diagnostic groups. The number of comorbid diseases did not predict SRH.

**Conclusion:**

Emotional well-being and physical health appraisal were strong predictors of SRH. Thus, SRH may be improved by influencing well-being and physical health appraisal via targeted interventions.

## Introduction

The importance of self-rated health (SRH) for various health-related outcomes such as morbidity and mortality is well documented (1, 2). SRH is defined as “a summary statement about the way in which numerous aspects of health, both subjective and objective, are combined within the perceptual framework of the individual respondent” [(3), p. 93]. The importance of SRH was demonstrated initially by Mossey and Shapiro who reported that it was a strong predictor of mortality, especially in the elderly, and even after controlling for potential confounding variables (4). Since then SRH has frequently been shown to predict various health-related outcomes (5–11).

**Figure 1:**
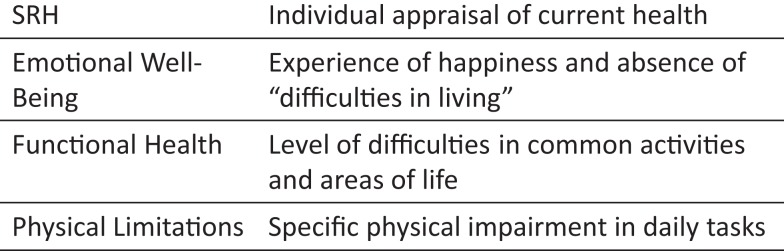
**Short definition of key constructs**.

The association between SRH and key outcomes like morbidity and mortality has not been clarified, yet. More information about SRH is required to explain its effect on morbidity and mortality. As SRH cannot be improved directly the determinants are our only means to indirectly affect the important measure of SRH. In order to improve SRH, we have to understand, which variables affect SRH to what extent and how these mechanisms differ for example between different diseases. The knowledge of how people rate their health and the health aspects involved may lead to intervention strategies to improve (self-rated) health. Short definitions of the key constructs for the following analyses are displayed in Figure 1.

Health- and lifestyle-related determinants of SRH have been addressed in a few studies [e.g., see Ref. (12–18)]. In order to understand the concept of SRH the influencing role of functional health might be particularly important. A recent study showed the key role of functional health status for SRH especially in the elderly (19). Functional health is often used synonymously with health-related quality of life as both concepts describe the functional ability to perform daily tasks. This ability might be reduced due to symptoms of a disease and the corresponding physical limitations. The emotional consequences of functional limitations (such as pain, depression, or anxiety) are related to the concept of functional health as well. Emotional well-being is considered to be another important predictor of SRH. It is often also referred to as psychological or mental well-being and reflects the subjective experience of happiness (20). Emotional well-being has been shown to be positively associated with SRH especially in the elderly (21). In a large meta-analysis emotional well-being was identified as a significant determinant of physical health (22). Perrucio et al. (2012) reported that it is not medical comorbidity alone that contributes to health perceptions but different aspects of functional and mental health (14). Steinhauser et al. compared health-related factors between three life-limiting diseases [cancer, chronic heart failure (CHF), and chronic obstructive pulmonary disease (COPD)]. They showed that functional limitation was more dependent on the specific disease than on the individual, whereas the other health-related factors did not differ between diseases but between subjects (18). Hence, the severity of functional limitations differentiates between diseases when other health-related factors are comparable. Nutzel et al. (23) confirmed the importance of disease-dependent health appraisal for multimorbid patients. They showed that the specific consequences of diseases in terms of limited functional ability and well-being were associated with SRH. Furthermore, Schuez et al. (13) concluded in their study that predictors of SRH varied depending on individual physical health: with increasing number of diseases the association of SRH with functional health increased. Thus, functional limitations and well-being are key concepts in understanding individual health appraisal especially in chronically ill individuals.

Consequently, in order to understand and possibly change SRH, it is crucial to identify and explain the factors that have an impact on it. Research findings so far suggest that people base their SRH on different aspects of health that depend on the individual appraisal. Thus, SRH might not directly depend on comorbidity but on specific appraisal that may be associated with comorbidity.

These associations have not yet been investigated within a larger sample of multimorbid individuals. Thus, in the current study the effects of health appraisal and comorbidity on SRH are analyzed longitudinally in three large multimorbid samples of persons with diabetes, CHF, or both diseases combined.

## Hypotheses

We expect former SRH to be the strongest predictor of future SRH. Additionally, future SRH is also predicted by a number of variables that reflect *health appraisal* such as general well-being, functional health, and physical limitations (hypothesis 1). Further, within the association of former and future SRH, the number of comorbid diseases has no additional mediating effect when measures of health appraisal are included into the model (hypothesis 2).

## Materials and Methods

### Participants and Procedure

From a pool of insurants of a German health insurance company (Techniker Krankenkasse), a sample of individuals suffering from diabetes, CHF, or both conditions [double diagnosis (DD)] was randomly selected and asked to answer self-report questionnaires regarding their health. The data collection was conducted and ethically approved by the insurance company. The participants did not participate in any intervention over the course of the data collection. The questionnaires were sent by mail to the participants by the insurance company. Individuals who did not suffer from dementia or severe mental diseases and provided written informed consent were included in the study. The already processed and de-identified data were transmitted to the authors and analyzed according to a pre-assigned study-protocol. At the two assessment points, T1 (baseline) and T2 (after four months), participants were asked to evaluate their SRH, general well-being, functional health as well as their physical limitations. During the 4 months, they continued to receive their usual medical treatment. Of initially, *n* = 3706 persons (*n* = 1240 with diabetes, *n* = 1229 with CHF, *n* = 1237 with DD) a subsample of *n* = 2863 (77.25%) participants returned both questionnaires at time 1 and time 2 [*n* = 974 (78.50%) with diabetes; *n* = 955 (77.71%) with CHF; *n* = 934 (77.51%) with DD]. Current analyses are based on these samples.

Table 1 presents the sociodemographic characteristics of the three groups. On average participants were 67.9 years old, the proportion of male participants was high (about 80%). All individuals in our study were suffering from chronic diseases that imposed a great burden on their lives. On average they had 4.9 comorbid diseases (like COPD, cancer, arthritis or coronary heart disease). Their SRH, emotional well-being, functional health, and physical limitations were in medium range and did not change significantly during assessment time.

**Table 1:** **Sample description**.

	**Diabetes**	**CHF**	**DD**

*n*	974	955	934
Gender (female %)	18.8	21.3	21.2

	**Mean (SD)**	**Mean (SD)**	**Mean (SD)**

Age	67.27 (7.51)	67.07 (8.77)	69.28 (7.02)
Number of comorbid diagnoses	4.26 (1.24)	4.23 (1.33)	5.50 (1.35)
SRH at T1	5.46 (1.98)	5.63 (1.90)	5.08 (2.00)
SRH at T2	5.59 (1.95)	5.71 (1.97)	5.09 (1.96)
Well-being at T1	58.16 (24.49)	60.26 (23.11)	54.03 (25.02)
Well-being at T2	58.84 (24.48)	60.88 (23.20)	55.65 (24.86)
Functional health at T1	0.78 (0.24)	0.83 (0.20)	0.74 (0.25)
Functional health at T2	0.78 (0.24)	0.83 (0.21)	0.74 (0.26)
PLS at T1	73.30 (24.19)	74.20 (22.44)	63.62 (25.05)
PLS at T2	68.74 (27.72)	70.44 (25.71)	59.46 (27.95)

*CHF, chronic heart failure; DD, double diagnosis; SRH, self-rated health; PLS, physical limitation score*.

*Higher scores indicate better well-being, better functional health, and less physical limitations*.

### Measures

Self-rated health, emotional well-being, functional health, and physical limitations were assessed at T1 (baseline) and T2 (4 months later) by questionnaires. Diagnoses, age, and gender were derived from insurance data at baseline.

Self-rated health was assessed using a well-established single-item measure [e.g. see Ref. (7, 24)]. Participants were asked to estimate their SRH on a scale ranging from 0 to 10. The exact wording of the single-item measure was: “If you were to rate your general state of health on a scale from 0 to 10 – (“0” meaning “couldn’t be worse” and “10” meaning “couldn’t be better”) – how would you rate your current state of health?” Research results and psychometric properties concerning this specific measure are described elsewhere (25, 26).

#### Functional Health and Physical Limitations

##### Functional Health

The EQ-5D questionnaire was used to assess functional health (27). Based on the five subscales, *mobility*, *anxiety/depression*, *usual activities*, *pain/discomfort*, and *self-care* and the level of difficulties were summarized in global scores, which were computed according to the EQ-5D value sets (28). For Germany, global scores range between −0.207 and 1, where −0.207 indicates “severe problems on all dimensions” and a score of 1 indicates “no problems on any dimension.” Hence, larger scores indicate a better functional health. Several studies [e.g., see Ref. (29–31)] evaluated psychometric characteristics of the EQ-5D in various samples and different diseases and found moderate to high retest-reliability scores (*k* = 0.67–0.85). Also, construct (29–31) and criterion validity (30) of the EQ-5D were consistently good. All authors conclude that the EQ-5D generates valid and reliable evaluation of functional health.

##### Physical Limitations

In order to assess perceived physical limitations in terms of impairment in daily activities, the physical limitation subscale from Kansas City Cardiomyopathy Questionnaire [KCCQ; (32, 33)] was applied. The physical limitations subscale assesses how much a patient’s condition affects his functional ability to do the following seven activities over the past 2 weeks: “dressing yourself,” “showering/bathing,” “walking 1 block on level ground,” “doing yardwork or housework,” “carrying groceries,” “climb a flight of stairs without stopping,” and “hurrying or jogging as if to catch a bus”. Participants responded on a 5-point scale from “extremely limited” to “not at all limited” with the additional option “limited for other reasons or did not do the activity.” According to the KCCQ-scoring instructions, if at least three of the questions were not missing, a mean score of the actual responses was transformed to a 0–100 scale with 0 meaning “extremely limited on all measured dimensions” and 100 meaning “no limitations on the measured dimensions”. The KCCQ shows good internal consistency (*Cronbach’s alpha* > 0.70) and satisfactory external validity (34, 35), especially the physical limitation scale used in the current study is a reliable measure [Cronbach’s alpha = 0.85, Ref. (34)].

#### Well-Being

To assess well-being, we used the World-Health-Organization-Five scale (WHO-5), a brief and commonly used measure of emotional well-being [e.g., See Ref. (36)] ranging from 0 to 100. Higher scores indicate better well-being. The WHO-5 has shown excellent internal consistency (*Cronbach’s alpha* = 0.91) and good external validity against SCID (Structured Clinical Interview for DSM Disorders; depression rating agreement of 80%) (37, 38). Also, the comparative validity with physicians’ diagnoses is reported to be excellent. While physician sensitivity for detecting major depressive disorder was only 40%, WHO-5 screening identified 94% of patients with major depressive disorder (37). According to the WHO, a score <52 indicates poor emotional well-being, and a score <28 is regarded as an indicator of a major depressive disorder (38).

#### Comorbidity

The number of comorbid diseases was calculated from insurance data by summing up all diagnoses from a list of 11 diseases that a participant had been diagnosed with in the previous 12 months before participation (e.g., arthrosis, coronary heart disease, CHF, COPD, diabetes).

### Data Analysis

Data from all participants who returned both questionnaires at time 1 and time 2 (*n* = 2863) were included in the analyses.

**Figure 2:**
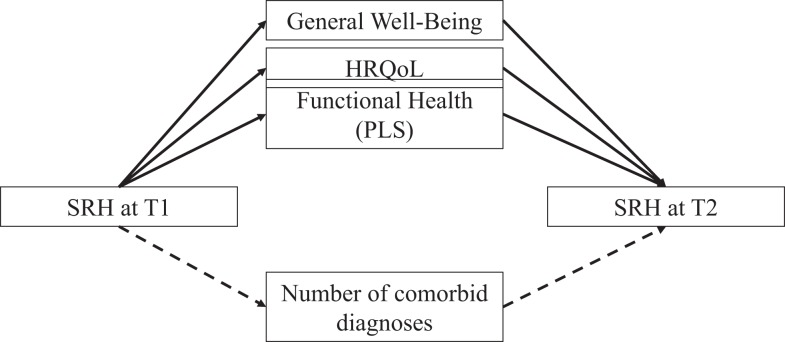
**Proposed longitudinal multiple mediator model**.

To test multiple mediation for the three subsamples (diabetes, CHF, and DD), the following longitudinal multiple mediator model was proposed (Figure 2).

Self-rated health at T1 was the focal predictor of SRH at T2. The number of comorbid diagnoses was included as a first mediator into the model. Further proposed mediators were emotional well-being, functional health, and perceived physical limitations. The mediator analysis was conducted separately for the three groups (diabetes, CHF, and DD). Age and gender were entered as covariates into the model. To compare the magnitudes of the indirect effects, pairwise contrasts were estimated.

The traditional way to conduct a mediation analysis is the causal steps approach by Baron and Kenny (39). This approach imposes a number of difficulties, most importantly the inability to quantify the relative magnitude of the mediating effect as well as its significance. Therefore, current recommendations for testing mediation hypotheses use the product-of-coefficients-approach in line with bootstrapping strategies to obtain a bootstrapped estimate of the indirect effects and to deal with the rarely achieved assumption of multivariate normality (40). As this approach allows to include several mediators in one model, the comparison of specific indirect effects is possible and their magnitudes are quantifiable. In multiple mediator models, a specific indirect effect through a mediator might not be the same as the indirect effect of this mediator in a single mediator model because the mediators are likely to be intercorrelated. Therefore, the specific indirect effect in a multiple mediator model has to be seen as the ability of a mediator to mediate the effect of the predictor on the outcome within a set of other mediators in the model (40). The current statistic procedure involved bootstrapping analyses with 5000 bootstrap samples. For each mediating effect a sample was drawn 5000 times with replacement from the original sample and the mediating effect was computed. The resulting 5000 estimates were averaged to a bootstrapped estimate for the specific indirect effect. Confidence intervals of bootstrapped estimates are asymmetrically distributed, therefore they have to be corrected [e.g., See Ref. (41, 42)]. Thus, bias-corrected and accelerated 95% confidence intervals are reported in the current study.

Statistical analyses were conducted using IBM SPSS Statistics Version 20. To test multiple mediator models the SPSS macro provided by Preacher and Hayes (40) was used.

## Results

### Sample Characteristics

Table 1 presents the sample characteristics of the three groups. Individuals in the diabetes or CHF only groups were slightly younger (mean of 67.3 and 67.1 years) than those in the double diagnosis group (mean of 69.3 years). In contrast to most studies in clinical research, about 80% of the sample was male. This is due to the insurants’ structure of the Techniker Krankenkasse, where most of the older insurants worked in engineering, technical and therefore male-dominated fields of work. Results of a one-factor-ANOVA showed that the double diagnosis group differed significantly from the single diagnosis groups with consistently poorer health ratings on all measures at both assessment points. At baseline and at follow-up, persons with diabetes reported significantly lower functional health than persons with CHF (0.78 vs. 0.83; *p* < 0.001). Other than that the single diagnosis groups do not differ significantly on any other of the health-related measures at baseline or follow-up.

Table 2 shows the results of the correlation analyses. All proposed mediator variables (except number of comorbidities) were highly intercorrelated.

**Table 2:** **Correlations of predictors in the three diagnostic groups**.

		Number of comorbidities	SRH at T1	SRH at T2	Well-being	Functional health
Diabetes	SRH at T1	−0.142**				
	SRH at T2	−0.139**	0.670**			
	Well-being	−0.136**	0.566**	0.533**		
	Functional health	−0.128**	0.566**	0.483**	0.527**	
	PLS	−0.186**	0.479**	0.497**	0.480**	0.533**
CHF	SRH at T1	−0.110**				
	SRH at T2	−0.106**	0.666**			
	Well-being	−0.061*	0.561**	0.464**		
	Functional health	−0.132**	0.537**	0.478**	0.465**	
	PLS	−0.169**	0.579**	0.543**	0.452**	0.568**
DD	SRH at T1	−0.134**				
	SRH at T2	−0.118**	0.675**			
	Well-being	−0.109**	0.555**	0.460**		
	Functional health	−0.137**	0.543**	0.498**	0.492**	
	PLS	−0.189**	0.540**	0.447**	0.486**	0.538**

*p < 0.05.

**p < 0.01.

*PLS, physical limitation score; SRH, self-rated health*.

### Multiple Mediator Analyses

Results for all three multiple mediator analyses are presented in Table 3. To test whether comorbidity and health appraisal (emotional well-being, functional health and physical limitations) contributed to the effect of former SRH on future SRH multiple mediator models were tested. For all three subgroups, there was a significant total, direct, and indirect total effect demonstrating a partial mediation by the proposed variables (hypothesis 1). Specific indirect effects revealed no mediating effect of the number of comorbid diagnoses in any of the tested models (hypothesis 2).

**Table 3:** **Mediation models**.

	Diabetes				CHF				DD			
	Coeff.	SE	*p*		Coeff.	SE	*p*		Coeff.	SE	*p*	
Total effect	0.65	0.03	<0.001		0.69	0.03	<0.001		0.68	0.03	<0.001	
Direct effect	0.46	0.04	<0.001		0.48	0.04	<0.001		0.55	0.04	<0.001	

	**Bootstrapped point estimate**	**BCa 95% CI**			**Bootstrapped point estimate**	**BCa 95% CI**			**Bootstrapped point estimate**	**BCa 95% CI**		
		**SE**	**LL**	**UL**		**SE**	**LL**	**UL**		**SE**	**LL**	**UL**

Indirect effects												
Total	0.18*	0.03	0.14	0.24	0.21*	0.03	0.14	0.27	0.13*	0.03	0.07	0.18
Number of diagnoses	0.00	0.01	−0.01	0.01	0.00	0.00	−0.00	0.00	0.00	0.00	−0.00	0.01
Well-being	0.09*	0.02	0.05	0.14	0.07*	0.02	0.02	0.11	0.04*	0.02	0.00	0.08
Functional health	0.01	0.02	−0.03	0.06	0.06*	0.02	0.02	0.11	0.07*	0.02	0.03	0.11
PLS	0.08*	0.02	0.04	0.13	0.08*	0.03	0.01	0.13	0.01	0.02	−0.03	0.11
Pairwise contrasts												
Number of diagnoses vs. well-being	−0.09*	0.02	−0.14	−0.05	−0.07*	0.02	−0.11	−0.02	−0.04	0.02	−0.08	−0.00
Number of diagnoses vs. functional health	−0.01	0.02	−0.06	0.03	−0.06*	0.02	−0.11	−0.02	−0.07*	0.02	−0.11	−0.03
Number of diagnoses vs. PLS	−0.07*	0.02	−0.13	−0.03	−0.08*	0.03	−0.14	−0.01	−0.01	0.02	−0.05	0.04
Well-being vs. functional health	0.08*	0.03	0.01	0.15	0.01	0.03	−0.07	0.07	−0.03	0.03	−0.09	0.03
Well-being vs. PLS	0.02	0.04	−0.06	0.08	−0.01	0.04	−0.09	0.06	0.03	0.03	−0.04	0.09
functional health vs. PLS	−0.06	0.04	−0.14	0.01	−0.02	0.05	−0.10	0.07	0.06	0.03	−0.00	0.12

*Multiple mediation models: independent variable: SRH at T1; dependent variable: SRH at T2; mediators: number of diagnoses, well-being, functional health, PLS*.

*p < 0.05; adj. *R^2^* in diabetes: 0.48; adj. *R^2^* in CHF: 0.49; adj. *R^2^* in DD: 0.51.

*CHF, chronic heart failure; DD, double diagnosis; BCa, bias corrected and accelerated; CI, confidence interval; LL, lower limit; UL, upper limit; PLS, physical limitation score*.

Furthermore, analyses showed strong effects of the health-related mediator variables (functional health and physical limitations) that differed between the diagnostic groups.

#### Diabetes

Multiple mediator analysis revealed that the total indirect effect of SRH at T1 on SRH at T2 through the proposed mediators was significant [0.18, *BCa* 95% *CI* (0.14;0.24)] with an explained variance of *adj. R^*2*^* = 0.48. The examination of the specific indirect effects showed that for persons with diabetes (controlling for all other mediators) only well-being and physical limitations were significant mediators of the relationship between SRH at baseline and SRH at follow-up. No other variable contributed significantly to the indirect effect. To compare the magnitudes of the indirect effects, pairwise contrasts were estimated. The indirect effects’ magnitudes of physical limitations and well-being on SRH at T2 were significantly larger compared to the number of comorbidities (see pairwise contrasts in Table 3). Also, the magnitude of the indirect effect of well-being was significantly larger than the magnitude of the indirect effect of functional health on SRH. Physical limitations and well-being were equal in size in terms of their magnitude indicating a comparable mediating effect in the proposed mediator model.

#### Chronic Heart Failure

For the leading diagnosis, CHF multiple mediator analysis also showed a significant total, direct, and total indirect effect [0.21, *BCa* 95% *CI* (0.14;0.27)], indicating partial mediation of the proposed mediators taken as a set (*adj. R^*2*^* = 0.49). Each mediator alone (except number of diagnoses) also had a significant mediating effect over and above the SRH autoregression. Thus, well-being, functional health, and physical limitations, specifically mediated the association of former and future SRH in this diagnostic group. The magnitudes of the specific indirect effects via well-being, functional health, and physical limitations were significantly larger than the effect via number of diagnoses. The three measures of health appraisal did not significantly differ in strength compared to each other. Thus, their mediating effects in the proposed model can be regarded as similar.

#### Double Diagnosis

For persons with both diagnoses (diabetes and CHF) the multiple mediator analysis also revealed significant total, direct, and total indirect effects [0.13, *BCa* 95% CI (0.07;0.18)] indicating a partial mediation of the mediators taken as a set (adj. *R^*2*^* = 0.51). Only well-being and functional health showed significant specific indirect effects. Physical limitations and the number of diagnoses did not have a specific mediating effect over and above the SRH autoregression, well-being, and functional health. Pairwise contrasts showed significant differences in the strengths of the indirect effect only for functional health compared to the number of diagnoses, indicating that the indirect effect via functional health was stronger than the effect via number of diagnoses. The specific indirect effects of the other mediators were not significantly different from each another.

## Discussion

The findings confirm that emotional well-being, functional health and physical limitations partially mediate the association between former and future SRH. These mediators contributed to SRH differently in each group (see below). The number of comorbid diseases did not predict SRH beyond the proposed mediators.

Across all groups emotional well-being consistently mediated the effect of former on future SRH. The mediation via physical limitations was significant for diabetes and CHF but not for the DD group. Functional health mediated the effect on SRH for persons with CHF and those with DD but had no mediating effect for persons with diabetes.

Between-groups differences in determinants of SRH appeared in terms of specific health appraisal indicators such as functional health and physical limitations. While physical limitations reflect the specific functional ability in daily activities, functional health gives a broader perspective on health appraisal as it additionally considers experiencing anxiety and depressive symptoms as well as pain and discomfort. Regarding health appraisal, persons with diabetes based their health rating rather on the limited ability to perform daily activities (physical limitations). These findings confirmed recent results that showed the strong association of a decrease in functional health and a declining physical health in diabetes (43). Persons with CHF additionally considered functional health in order to self-rate their health. For the most affected group with the double diagnosis (diabetes and CHF combined) functional health but not physical limitations contributed to SRH beyond former SRH and emotional well-being. When functional health appraisal and well-being were included into the model, the number of comorbid diseases had no mediating effect anymore. Thus, it seems to be the appraisal of comorbidity and not the mere number of diseases that affects SRH.

Previous research has shown that individuals with poorer health reported stronger associations of SRH with functional status (13, 15, 16). In those studies, functional health was also assessed as the ability to perform daily activities, thus as a specific task-oriented measure like the current physical limitations measure. However, our results revealed different effects of functional health and physical limitations on SRH. The less healthy individuals in the current study (double diagnosis) based their self-rating of health mostly on functional health aspects whereas the individuals with a comparatively better health (single diagnosis of diabetes or CHF) seemed to base their self-rating more on the specific aspects of physical limitations.

Within the DD-group, the proposed model had the largest amount of explained variance (*R^*2*^* = 0.51) of future SRH mainly because of the large direct effect of former SRH. The direct effects in the diabetes and the CHF group were small compared to the DD-group but with larger indirect effects indicating a greater impact of the proposed mediators for single leading diagnoses. The considerably greater direct effect in the DD group suggests that individuals with both diagnoses based their self-rating more on former SRH than individuals with a single diagnosis. This finding should be examined further as it indicates a mechanism in the establishment of SRH depending on the appraisal of comorbidity. Heller et al. (17) concluded that younger individuals and those with low comorbidity were more likely to reduce self-ratings of health following changes in diagnosis than older persons with higher comorbidity. Hence, low comorbidity might be associated with greater cognitive flexibility in health appraisal. Therefore, research should focus on self-efficacy, control-beliefs, and cognitive flexibility associated with comorbidity to reveal further mechanisms of SRH constitution. Also, the question when which aspects of health become relevant for health appraisal should be addressed. Are the currently most limited abilities taken into account when persons self-rate their health?

Although our results indicate independence of SRH from the number of comorbid diseases, comorbidity is a strong predictor of mortality and should not be underestimated. However, research should focus on the processes behind the strong relationship of comorbidity and mortality. The individual *appraisal* of comorbidity might be more relevant than the simple number of comorbid diseases.

## Summary

Our data confirm that SRH cannot be captured as a stable construct with fixed determinants and that it is highly dependent on individual appraisal of physical health. The results show different determinants of SRH depending on diagnosis. However, emotional well-being was a consistent and powerful factor of SRH across all groups. Thus, by improving emotional well-being SRH may be enhanced. Future SRH depended strongly on former SRH but the magnitude of this effect differed between diagnostic groups. The effect was stronger for persons with a greater health burden (double diagnosis vs. single diagnosis). For comparatively healthier individuals (single diagnosis) the indirect effect via the proposed mediators was larger than for less healthy individuals (double diagnosis). That, again, suggests different mechanisms underlying health appraisal that depend on physical health. Also, in terms of specific health appraisal less healthy individuals seem to base their self-ratings of health rather on functional health including affective aspects, whereas healthier persons (single diagnosis) focus on specific aspects of physical limitations when they self-rate their health.

## Limitations

Unfortunately, we did not have the data to compare our results with a healthy sample. Future research should address potential differences in the constitution of SRH between different diagnostic patterns including healthy individuals.

Another limitation of our study is that due to the drop out over time the analyzed sample differed from the original sample. For methodological reasons, we included only those participants into the analyses that returned the second questionnaire (T2). Therefore the analyses are based on 77% of the baseline sample, which reflects our very high response rate. To test whether the participants who returned both questionnaires differed at baseline from those who returned only one, we ran an ANOVA. Results showed significant differences on all proposed variables consistently in the same direction. Compared to the participants who had to be excluded, the final sample reported higher SRH (5.39 vs. 4.9; *p* < 0.001), higher well-being (57.56 vs. 52.28; *p* < 0.001), better functional health (0.79 vs.0.74; *p* < 0.001), and less physical limitations (70.43 vs. 66.86; *p* < 0.001). Therefore, participants who were included into the analyses were significantly *less* affected in terms of health appraisal but had a slightly significantly higher number of comorbid diseases (4.65 vs. 4.54, *p* = 0.045) than those who could not be included into the analyses. We can only suspect that it might have been the even higher disease burden in terms of health appraisal that was responsible for non-participation due to higher physical/mental distress or less self-efficacy. In several studies with the health insurance company, we noticed a very high response rate (26, 44, 45). We assume that a reminder letter that was sent 2 weeks after the initial questionnaire added to this excellent response rate. Nonetheless, our results might be slightly biased because the overall health in the current sample was better than in the original sample.

Furthermore, the percentage of male participants (about 80%) was rather high. That allows us to supply information especially on men but also reduces the degree to which the results can be generalized.

As shown in Table 2, variables were highly correlated, which can compromise the significance of the effects found in the analyses. Especially the indirect effects might be biased due to intercorrelation between the measures. Regarding the topic of correlated mediators Preacher and Hayes (40) state that “… an intervention is sometimes designed to impact multiple intervening variables to achieve a desired outcome. In such cases, the mediators are almost necessarily correlated by virtue of their mutual reliance on a common cause…”. This conceptual similarity of the mediators may in part explain the strong correlations between the measures, however, it should be noted that all correlations were <0.6, indicating a substantial amount of discriminant validity.

## Conclusion

Self-rated health is a highly individual rating. It is influenced by emotional well-being, functional health, and perceived physical limitations. Emotional well-being affected SRH consistently across all disease groups in our study. Functional health and physical limitations differed in their effect on SRH depending on diagnostic group. SRH might have a different meaning in different diagnoses or combinations of diagnoses. Future research should focus on individual appraisal of comorbidity, compare disease patterns of different diseases, and also address healthy individuals to answer the question when which health aspects are taken into account to rate one’s own health. By affecting emotional well-being we might be able to improve SRH.

## Ethical Approval

The authors assert that all procedures contributing to this work comply with the ethical standards of the relevant national and institutional committees on human experimentation and with the Helsinki Declaration of 1975 as revised in 2013. This study was carried out in accordance with the recommendations of the ethical guidelines of the health insurance company (Techniker Krankenkasse). Further ethics approval was not required as per German ethical guidelines. The individuals in the current study provided written informed consent, and they did not participate in any intervention over the course of data collection.

## Conflict of Interest Statement

The authors declare that the research was conducted in the absence of any commercial or financial relationships that could be construed as a potential conflict of interest.
